# SARS-CoV-2 viability on sports equipment is limited, and dependent on material composition

**DOI:** 10.1038/s41598-022-05515-1

**Published:** 2022-01-26

**Authors:** Thomas Edwards, Grant A. Kay, Ghaith Aljayyoussi, Sophie I. Owen, Andy R. Harland, Nicholas S. Pierce, James D. F. Calder, Tom E. Fletcher, Emily R. Adams

**Affiliations:** 1 Department of Tropical Disease Biology, Liverpool School of Tropical Medicine, Pembroke Place, Liverpool, L3 5QA UK; 2 Wolfson School of Mechanical, Manufacturing and Electrical Engineering, Loughborough University, Ashby Road, Loughborough, LE11 3TU UK; 3 England and Wales Cricket Board and National Centre for Sport and Exercise Medicine, Loughborough University, Loughborough, LE11 3TU UK; 4 Fortius Clinic, London, W1U 2EU UK; 5 Department of Bioengineering, Imperial College London, London, SW7 2AZ UK; 6 Department of Clinical Sciences, Liverpool School of Tropical Medicine, Pembroke Place, Liverpool, L3 5QA UK

**Keywords:** Microbiology, Environmental microbiology, Virology

## Abstract

The control of the COVID-19 pandemic in the UK has necessitated restrictions on amateur and professional sports due to the perceived infection risk to competitors, via direct person to person transmission, or possibly via the surfaces of sports equipment. The sharing of sports equipment such as tennis balls was therefore banned by some sport’s governing bodies. We sought to investigate the potential of sporting equipment as transmission vectors of SARS-CoV-2. Ten different types of sporting equipment, including balls from common sports, were inoculated with 40 μl droplets containing clinically relevant concentrations of live SARS-CoV-2 virus. Materials were then swabbed at time points relevant to sports (1, 5, 15, 30, 90 min). The amount of live SARS-CoV-2 recovered at each time point was enumerated using viral plaque assays, and viral decay and half-life was estimated through fitting linear models to log transformed data from each material. At one minute, SARS-CoV-2 virus was recovered in only seven of the ten types of equipment with the low dose inoculum, one at five minutes and none at 15 min. Retrievable virus dropped significantly for all materials tested using the high dose inoculum with mean recovery of virus falling to 0.74% at 1 min, 0.39% at 15 min and 0.003% at 90 min. Viral recovery, predicted decay, and half-life varied between materials with porous surfaces limiting virus transmission. This study shows that there is an exponential reduction in SARS-CoV-2 recoverable from a range of sports equipment after a short time period, and virus is less transferrable from materials such as a tennis ball, red cricket ball and cricket glove. Given this rapid loss of viral load and the fact that transmission requires a significant inoculum to be transferred from equipment to the mucous membranes of another individual it seems unlikely that sports equipment is a major cause for transmission of SARS-CoV-2. These findings have important policy implications in the context of the pandemic and may promote other infection control measures in sports to reduce the risk of SARS-CoV-2 transmission and urge sports equipment manufacturers to identify surfaces that may or may not be likely to retain transferable virus.

## Introduction

Public health interventions to control the COVID-19 pandemic in the UK have necessitated restrictions in social mixing, with both amateur and professional sports either prohibited, or allowed with considerable infection control measures in place^1^. It has been estimated that up to 44% of transmission occurs prior to symptom onset, when viral loads are highest^2^, meaning people are unaware of the infection and will continue in their daily activities, including sports participation. All team sport in the UK was postponed and subsequently cancelled from mid-March 2020 and although a staged return to elite sport was enabled from April^3^, there was concern that participation in community sport could risk disease transmission. Limited return in team sport with strict hygiene measures at sports grounds and rule changes for some contact sports were imposed.

The major risk of SARS-CoV-2 transmission during team sports is likely direct player to player transmission via respiratory droplets^4^, either during play or socially before and after the game. During non-contact sports such as cricket and soccer the risk of transmission is considered very low because only fleeting incursions of social distancing are seen^5^. Few amateur sporting events have been linked to SARS-CoV-2 transmission. A Danish study on professional football reported that during a 90 min match the average time any player spent within 1.5 m of another was 87.8 seconds^6^, and a study of the return of competitive football in Germany concluded that training and matches may be carried out safely during the SARS-CoV-2 pandemic^7^. Careful evaluation of elite level contact sports such as rugby failed to demonstrate evidence of transmission during the course of playing and training^8^.

Although there is uncertainty about the role of fomites in SARS-CoV-2 transmission^9^, equipment which is commonly shared in sports was considered as a potentially important route of transmission. In June 2020 the UK Prime Minister Boris Johnson stated in the House of Commons that cricket balls are a "natural vector" of coronavirus provoking a lively debate in the press on the safety of opening community sports. When team sports were re-introduced, so too were recommendations to reduce the sharing of sports equipment such as tennis balls. Whilst the viability of virus on a variety of surfaces has been demonstrated, transmission requires the deposition of virus onto a surface, then the transfer of enough virus to cause an infection from that surface to the mucus membranes of another person. Viral shedding into the environment has been demonstrated during SARS-CoV-2 infections, for example the rooms of hospitalised SARS-CoV-2 patients can be heavily contaminated with SARS-CoV-2, including frequently touched surfaces such as sinks and door handles^10^. This is thought to be from the spread of respiratory droplets via breathing, sneezing and coughing^11^, and the transfer of SARS-CoV-2 to objects from the hands of patients has been documented^12^. The minimum infectious dose of SARS-CoV-2 is currently unknown^13^, which makes quantifying the required viral load for transmission difficult.

It has been hypothesised that because physical sports lead to increased respiration, deep exhalation is likely to increase the expulsion of droplets or aerosols containing infectious particles which may contaminate sports equipment. Additionally, spitting is common in some team sports such as football and rugby and saliva has been used to shine cricket balls. Secretions from the upper respiratory tract have the potential to carry high viral loads^14^, which could then be transferred onto materials such as clothing or balls. Studies on viral stability on sporting equipment are limited. Pelisser et al. reported that inactivated SARS-CoV-2 may be detected by RT-qPCR from the surfaces of cricket balls up to one hour past the inoculation^15^, although viral viability could not be determined using this approach. Determining the potential for SARS-CoV-2 transmission whilst sharing sports equipment is crucial when producing guidelines to mitigate risks for return of community and elite team sports. This study [SARS-CoV-2 Transmission Risk from sports Equipment (STRIKE)] aimed to quantify the recoverable live virus from droplets of viable SARS-CoV-2 suspensions deposited onto a variety of sporting equipment, over a time frame relevant to sporting activity.

## Methods

### Materials

Sporting materials were selected from the materials collection at the Sports technology Institute at Loughborough University (Table 1). These included commonly handled objects from popular sports, such as rugby, football and tennis balls. All materials were unused prior to testing. Materials frequently shared between participants were prioritised due to a greater potential as transmission vectors. Materials were cut into 2 cm diameter disks using a metal hole punch. The red cricket ball was not cut into disks, and instead used whole, due to difficulties retaining surface integrity during removal. Materials were not sterilised prior to inoculation, so as not to affect the surface coatings, and antibiotics in the media were relied upon to avoid contamination during cell culture. Steel disks were purchased (Lasermaster, UK) for use as a control surface.

**Table 1:** Equipment and materials used in the study.

Equipment type	Standard	Surface material
Cricket glove (palm)	BS 6183-4: 2001*	Calf leather
Control	Stainless steel	
Football	FIFA Quality Pro. Law 2: IFAB Laws of the Game	Thermoplastic Polyurethane (TPU)
Golf ball	Part 4, The Equipment Rules, USGA. Named on list of conforming balls, USGA, R&A	SurlynTM ionomer resin
Gym pit foam	n/a	Polyurethane (PU) foam (open cell)
Horse saddle	n/a	Polyurethane (PU)
Red Cricket ball (unused)	BS 5993:1994* Law 4, The Laws of Cricket, MCC	Bovine leather burnished with synthetic grease
Rugby ball	Law 2: Ball, World Rugby Laws	Rubberised polyester (PES)
Tennis Ball	ITF Approved, Rule 3, ITF Rules of Tennis	Raised Wool/Nylon woven cloth
White Cricket ball (unused)	BS 5993:1994* Law 4, The Laws of Cricket, MCC	Bovine leather with nitrocellulose coating

*Indicates standards available to certify such products, however it is not known whether product was subject to standard testing.

### Material inoculation

Materials were inoculated with a 40 μl droplet of Dulebecco’s Modified Eagles Medium (DMEM) containing a high (5.4 × 10^4^ plaque-forming units (PFU)) or low (5.4 × 10^2^ PFU) concentration of quantified live SARS-CoV-2 virus (isolate REMRQ0001/Human/2020/Liverpool). Inoculum concentrations were chosen as representing the upper and lower quartile of viral loads in symptomatic patients^15^. All work with live virus took place under BSL3 conditions in a Class 2 biological safety cabinet. Materials were inoculated on the outward facing surface, and care was taken to ensure the inoculum did not run off the material during the inoculation. Triplicate pieces of each material were inoculated for the following time points: 1, 5, 15, 30, 90 min. At each time point the materials were swabbed using dry cotton swabs (Copan, Italy), and added to 400 μl of DMEM. A standardised swabbing technique was employed for each sample to reduce variation, with the swab being dragged upwards for two seconds and sideways for two seconds. The tubes containing the swabs were vortexed for 5 s, and then serially diluted ten-fold for three dilutions, in DMEM. During the study period the laboratory temperature and humidity were 22.1 °C ± 1.6 and 52% relative humidity ± 3.8%.

### Cell culture

Cultures of VERO E6 cells (C1008; African green monkey kidney cells, European Collection of Authenticated Cell Cultures 85,020,206) were maintained in T75 cell culture flasks (Corning, US) in Dulbecco’s Modified Eagles Medium (DMEM) supplemented with 4.5 g/L glucose and l-Glutamine (Lonza, US), 10% foetal bovine serum (Sigma, US) and 50 units per ml of penicillin/streptomycin (Gibco, US), at 37.5 °C + 5% CO2. Cells for plaque assays were detached from the monolayer using 2 ml 1 × trypsin–EDTA (Sigma, US) and 500 μl was seeded into 24 well microtitre plates (Corning, US) at a density of 250,000 cells/ml. Plates were incubated for 24 h at 37.5 °C + 5% CO_2_ and used for downstream plaque assays if judged to be > 95% confluent by microscopy.

### Viral plaque assays

Viral plaque assays were carried out using VERO E6 cells in 24 well microtitre plates. Media was aspirated from the microtitre plates, and 40 μl of media from the swabs and further dilutions were added to triplicate wells with 160 μl of DMEM 2% FBS. Microtitre plates were incubated for 1 h at 37.5 °C + 5% CO_2_ to ensure viral infection. Plates were then removed from the incubator and overlaid with a 1.1% suspension of cellulose (Sigma, UK) in DMEM 2% FBS, and incubated again under the same conditions for 72 h. Plates were then removed from the incubation, fixed with 100% formaldehyde for one hour, and stained with 1 ml/well of 0.25% crystal violet solution. After staining for 1 min, plates were gently washed with water, then air dried for > 3 h. Viral plaques were then visually counted for each well. The total PFU retrieved from the swab was then back calculated from the viral plaque counts from the 40 μl media tested. A schematic of the methods is shown in Fig. S1 (produced using BioRender, https://biorender.com/).

### Outcomes

All individual plaque counts for each material/time point swab were used to analyse the viral recovery from each material. Readings below the limit of quantitation (BLQ) were assumed to be equal to the BLQ levels/2. To characterise SARS-CoV-2 retained on different materials, a dynamic approach was used to measure the time-reduction course of virus. This was achieved by estimating viral decay half-life through linear models to log transformed PFU data on each material. The model assumed a single-phase decay profile on all materials over time. Each material was assumed to have a different intercept and slope as the materials varied widely in the observed initial levels of virus at the first minute.

### Statistical analysis

Linear fitting was achieved through the *lm* function in R. After generating a slope and intercept for each material, simulations were performed based on the estimates for each of these parameters and their covariance using R. 500 simulations were performed for each material and their 5–95 percentiles were plotted against the observed data. To estimate the overall exposure to virus over time for each material, the area under the curve (AUC) of the time-virus simulated profile was estimated for each of the 500 simulations and compared as the primary measure describing the overall exposure of each material to the virus over time. Simulated AUCs for each material were compared statistically using non-parameteric pairwise comparisons using Wilcoxon rank sum test. Materials were ranked based on AUC in comparison to the control material on all reported graphs. Simulated half-life and AUC data are presented as box and whiskers plots displaying the 5–95 percentiles of the 500 simulated profiles for each material.

### Patient and public involvement statement

As this was solely a laboratory study we did not involve any patients or members of the public in the study design.

## Results

**Figure 1:**
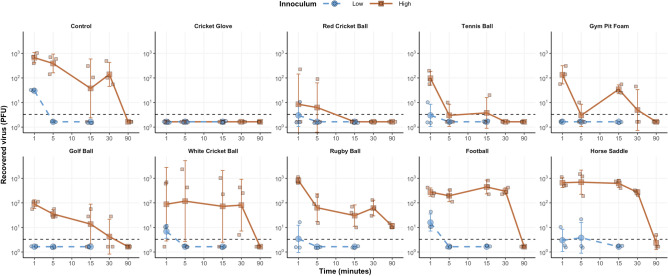
Recovered virus from all materials inoculated with 5.4 × 10^2^ PFU (low inoculum, blue line) and 5.4 × 10^4^ PFU (high inoculum, orange line) across the 90 min sampling time, including the control sample (steel disk). Large cirlcesrepresent the geometric mean of three replicates (shown as small circles) and Error bars indicate the geometric standard deviations.

**Figure 2:**
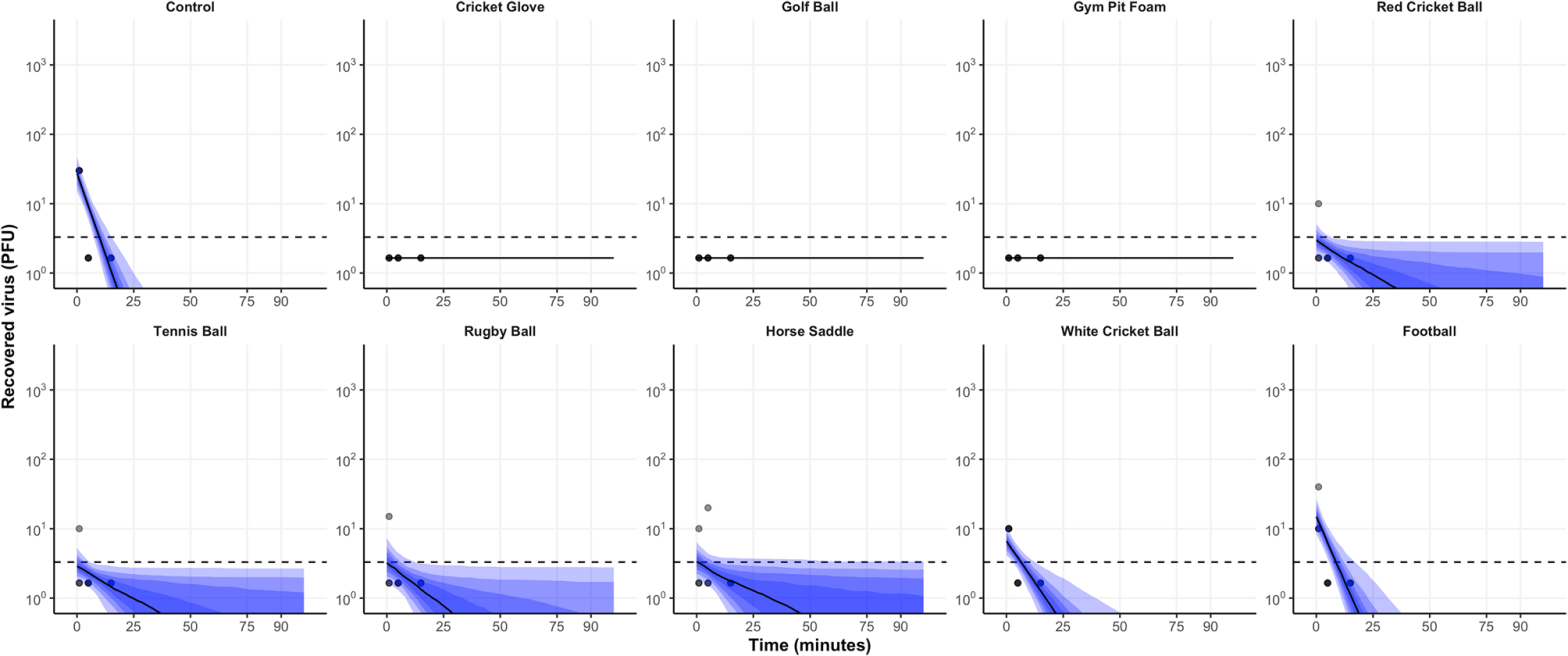
The predicted median decay of viral titres (solid black line) with 5–95 percentiles (shaded red areas) overlaid with observed PFU (grey circles) from all materials inoculated with 5.4 × 10^2^ PFU (low inoculum) using a linear regression model on log-transformed data.

**Figure 3:**
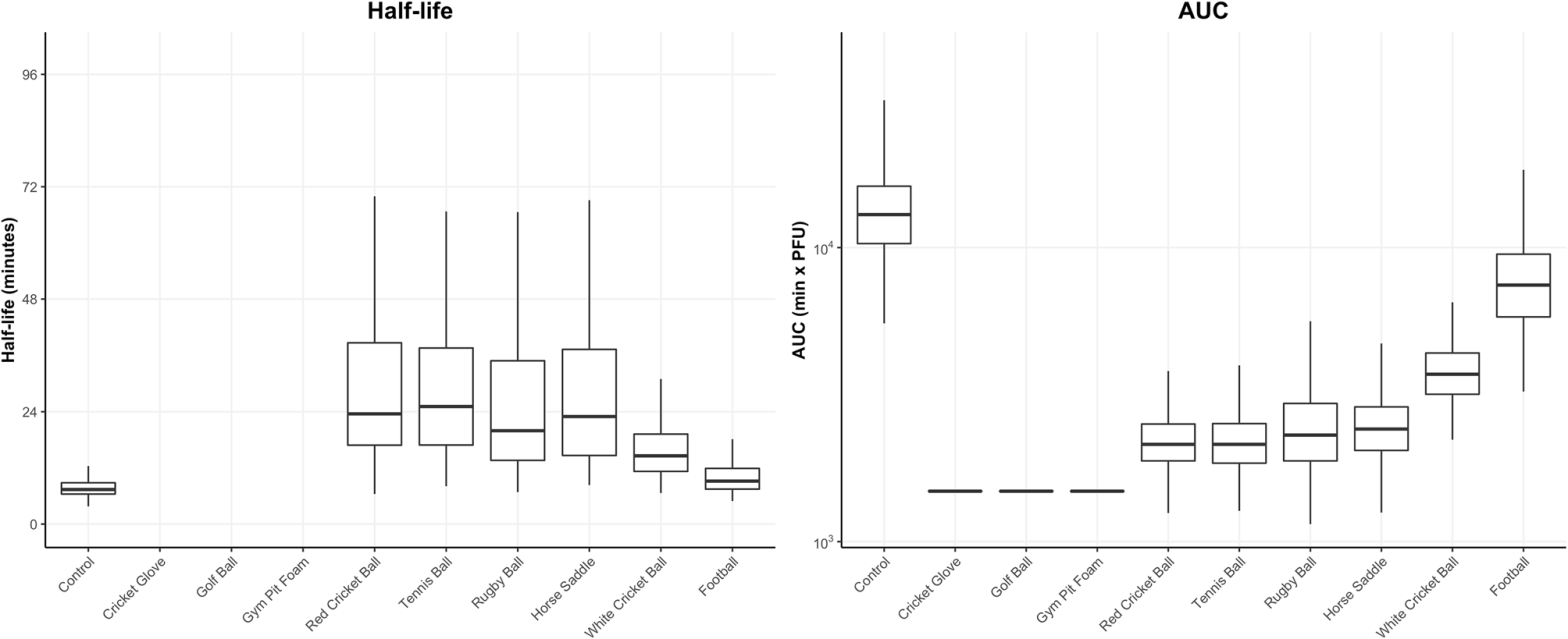
Box and whiskers plots representing the Half-life and area under the curve (AUC) distribution of 500 generated profiles for each material inoculated with SARS-CoV-2 at 5.4 × 10^2^ PFU (low inoculum).

At the one-minute time point SARS-CoV-2 was only detected in 7/10 materials when using the low inoculum dose (recovered virus ranging from 10 to 40 virions) (Fig. 1). At five minutes virus was detected on the horse saddle alone (20 virions) and no virus was detected on any material at 15 min. The model-predicted decay for the low inoculum is shown in Fig. 2 and the AUC and predicted half-life for the low inoculum were lower than that of the high inoculum (Fig. 3).

For the high inoculum dose, virus was recoverable from every material except the cricket glove at the one-minute time point (Fig. 1) but the viral titre reduced from 5.4 × 10^4^ to an average of 3.7 × 10^2^ (range of 1 × 10^3^ to 0) virions. The highest recovery was obtained from the rugby ball, steel control disks and horse saddle with the lowest viral titres retrieved were from absorbent materials such as the cricket glove, red cricket ball and tennis ball. Viral recovery reduced over time for all materials tested and no virus could be retrieved at 90 min, except for the horse saddle and rugby ball, although viral levels had reduced to 2 and 12 virions, respectively. The mean recovery reduced significantly at the one, five, 15, and 30-min time points for all materials (P = 0.0137, 0.0185, 0.0174, and 0.0117, respectively). The mean recovery of virus fell across all materials to 0.74% at one minute, 0.39% at 15 min and 0.003% at 90 min (Fig. S2).

**Figure 4:**
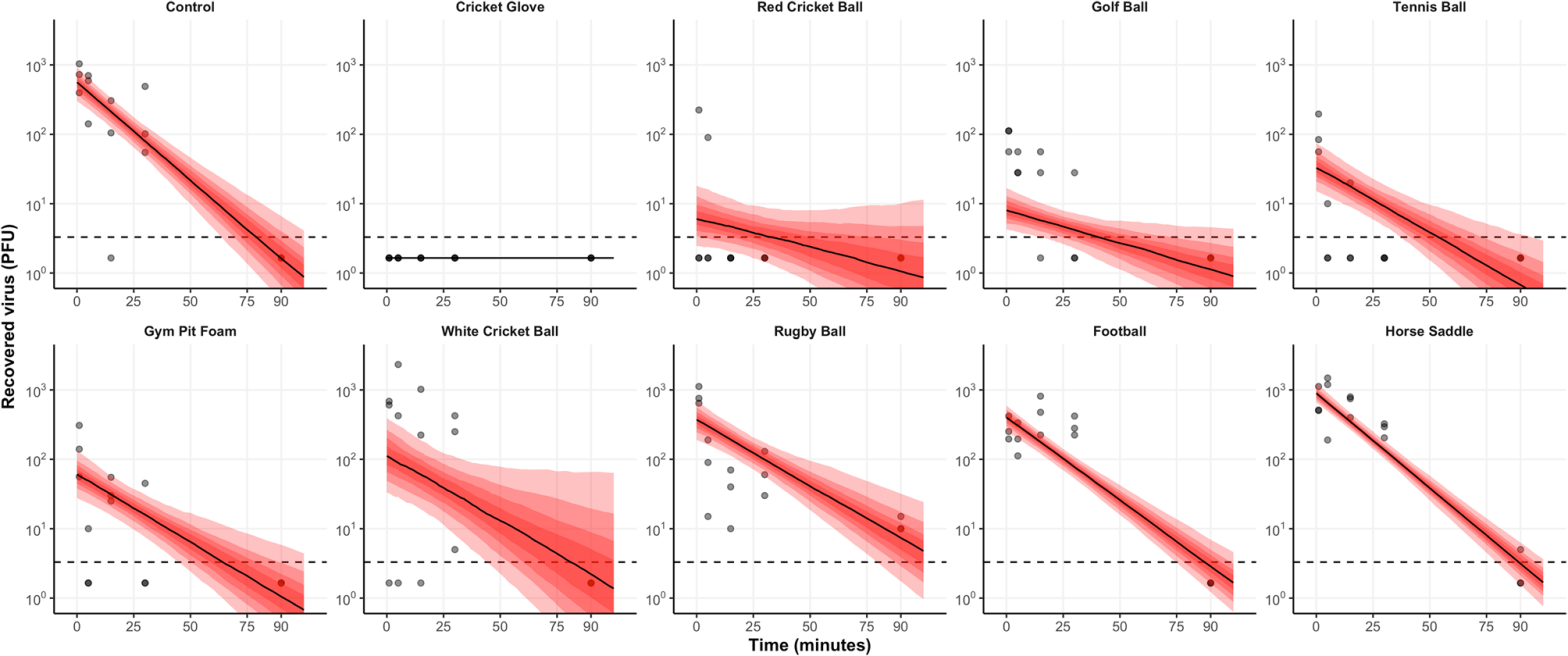
The predicted median decay of viral titres (solid black line) with 5–95 percentiles (shaded red areas) overlaid with observed PFU (grey circles) from all materials inoculated with 5.4 × 10^4^ PFU (high inoculum) using a linear regression model on log-transformed data.

**Figure 5:**
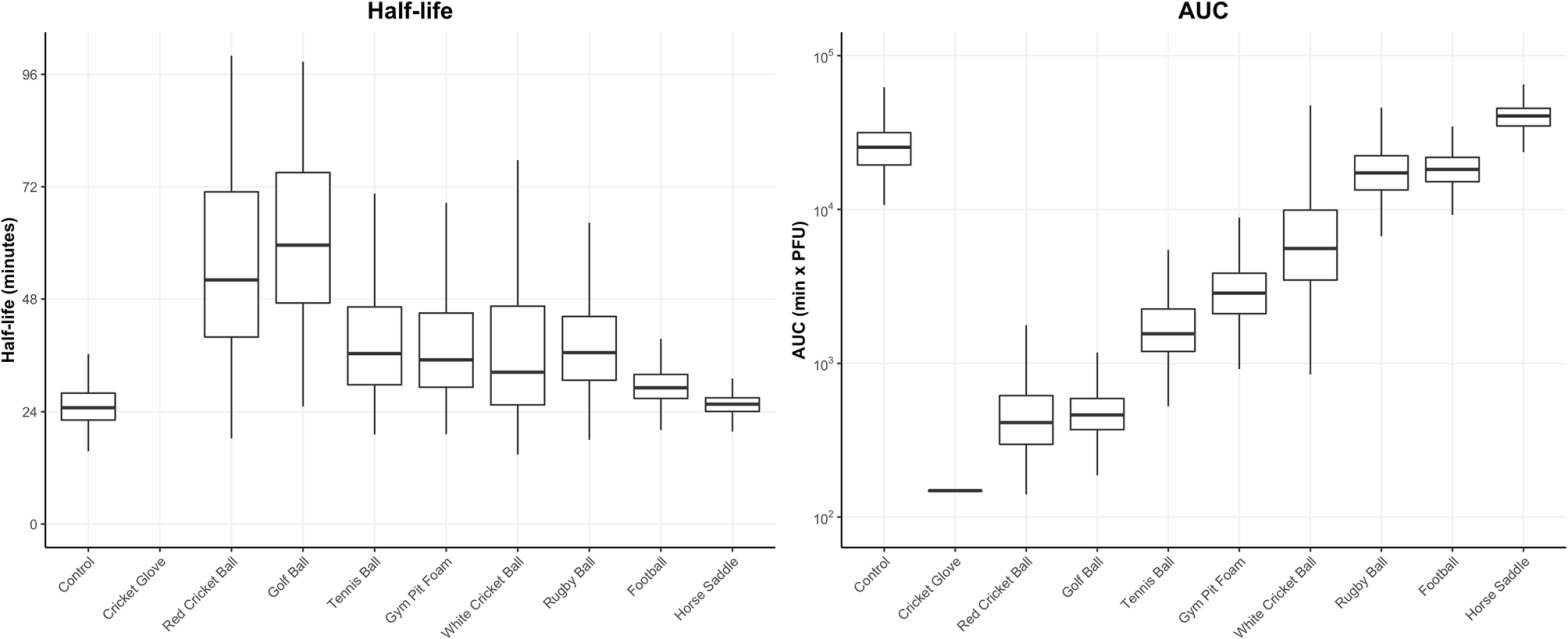
Box and whiskers plots representing the Half-life and area under the curve (AUC) distribution of 500 generated profiles for each material inoculated with SARS-CoV-2 at 5.4 × 10^4^ PFU (high inoculum).

Exponential viral decay was predicted from the linear regression models for all surfaces tested, indicated by a straight line of decay on the log^10^ viral PFU scale (Fig. 4). Data from the white cricket ball was more varied, with subsequent wider ranges in possible decay rates. The estimated mean half-life of deposited virus after the one-minute time point ranges from 24 min (control) to 60 min (golf ball) (Fig. 5). The area under the curve (AUC) analysis takes into account the initial decrease in recoverable virus and ranks the surfaces in terms of transmissibility of SARS-CoV-2, from porous materials such as the cricket ball to less porous materials such as the horse saddle. Analyses using Wilcox non-parametric test showed that AUCs simulated for different materials are statistically different with p < 0.001 with the horse saddle containing the highest amount of virus over time and the cricket glove the lowest.

## Discussion

This laboratory-based study demonstrates an exponential reduction in detectable live SARS-CoV-2 virions for all inoculated sports equipment over a short time period. The study focussed on materials that are likely to be shared or touched by multiple people during individual or team sports, including balls from a range of sports, plus cricket gloves, gymnastic spit foam, and a racing horse saddle. The low inoculum (representing droplets from the lower quartile of viral loads in symptomatic patients)^16^ could only be detected on the horse saddle at five minutes and no virus could be detected on any material at 15 min. 0.74% of virus was recoverable at one minute in the high inoculum (representing droplets from the higher quartile of viral loads), 0.39% at 15 min and just 0.003% at 90 min. This indicates that transfer of sufficient virus from fomites is unlikely from individuals with lower viral loads. As the viral inoculum dried over time, less virus was recoverable, matching previous studies on inanimate surfaces^17^.

The contribution of fomites to the transmission of SARS-CoV-2 is controversial^18^. Wilson et al.^19^ used a Monte Carlo simulation to perform a quantitative microbial assessment of the risk of infection from fomites, and found a lower than 1/10,000 infection risk from surfaces infected with a range of 1 to 10,000 genome copies/cm, supporting the potentially limited role of fomites in SARS-CoV-2 transmission. In addition, it should be noted that in certain sports, equipment such as balls may come in contact with crowds where unknown viral load may be encountered. Participants in sports are likely to be asymptomatic, however viral loads in this sub group appears to be similar to those in symptomatic patients^20^. A South Korean cohort study of 303 non-hospitalised SARS-CoV-2 positive patients (193 symptomatic/110 asymptomatic) found no significant difference in RT-qPCR Ct values between the groups^21^. Quantification of the viral load that may be transferred from an individual with SARS-CoV-2 infection onto sports equipment has not been evaluated but this study used previously reported concentrations seen in respiratory tract secretions.

### The effect of material composition on SARS-CoV-2 transfer

We found that viral recovery was reduced by absorbent materials such as leather (red cricket ball and cricket glove) and polyurethane foam (gym mat foam). Despite the white and red cricket ball surfaces both being composed of bovine leather, the different coatings used to finish the surfaces (synthetic grease on the red ball, nitrocellulose on the white ball) had a noticeable effect on viral recovery, with the red ball having a lower level. These properties were observed in unused cricket balls. It is expected that with use cricket balls will lose the coatings and may become more porous. Previous studies have shown that viruses such as avian influenza have shorter recovery times from porous materials or open-cell foam structures^22^, presumably as viral particles are trapped inside and are not easily transferred during contact. The physiochemical interactions between the viral capsid and the material are also likely to impact the viral viability and transfer from the material^23^, and therefore the hydrophobicity and electrostatic properties of polymer surfaces may be important in their role as fomites. The observation that porous materials result in reduced viral recovery and transmission risk can be used to prioritise materials for within-game cleaning or swapping, and focus cleaning efforts to reduce their effect on sporting events.

### Limitations

All experiments were carried under a single temperature and humidity, two parameters known to affect SARS-CoV-2 viability^24^. Sports are played under different conditions due to seasonality and whether they take place in or outdoors. A more accurate assessment would include these variables. The minimum infectious dose of SARS-CoV-2 remains unknown^13^, and this makes it difficult to extrapolate the amount of virus on a surface necessary for transmission. The prospect of future human challenge models may provide this data^25^.

Our methodology employed a dry swab to retrieve the virus from the surfaces, in order to best replicate the transfer onto a player’s body or clothing. Higher viral recovery, and possibly less variation between replicates, would have been achieved by directly adding media to absorb virus^26^ or by using a wet swab^27^. However, this would not replicate the real-world conditions that the experiments were designed to assess. The recovery rate of dry swabs varies, but has been estimated as 32–38% for recovering MS2 phage from steel surfaces, depending on the elution media^28^. Therefore, more virus is likely to be present on the materials than our results infer.

This was a laboratory study, and further in-game behavioural studies are required to show frequency of potential transmission events to quantify risk. In practice many items of sports equipment are not routinely handed from person to person but instead rub against or collide with implements, other parts of the body, the ground and other sports infrastructure, presumably further reducing viral load. This is illustrated by a study showing SARS-CoV-2 RNA was not detectable from inoculated cricket balls after wiping, rolling, or bouncing on the floor^15^. We tested un-used sports equipment for the study, and material surfaces may change after short- or long-term usage, potentially affecting viral adherence and surface transmission.

## Conclusions

This study demonstrates the rapid decay in transmissible SARS-CoV-2 virus on several types of sports equipment and given the uncertainty of the role of fomites in the transmission of virus it is likely that close contact with other players either during play or pre/post-match travel and socialising is more important as a mode of spreading the virus. This has implications for policymakers introducing control measures during the reopening of sports. The differences in transfer seen between types of sports equipment may also direct the engineering of materials that retain and absorb virus, as opposed to hydrophobic materials where viral transfer is greater.

## Supplementary Information

Supplementary Figures.
