# Supplementary material for: SARS-CoV-2 viability on sports equipment is limited, and dependent on material composition
Source: Sci Rep. 2022 Jan 26;12:1416. doi: 10.1038/s41598-022-05515-1 (PMC8791971; doi:10.1038/s41598-022-05515-1)
Supplement: Supplementary file 1 — Supplementary Figures. [file 41598_2022_5515_MOESM1_ESM.docx]

**Supplementary Figures**


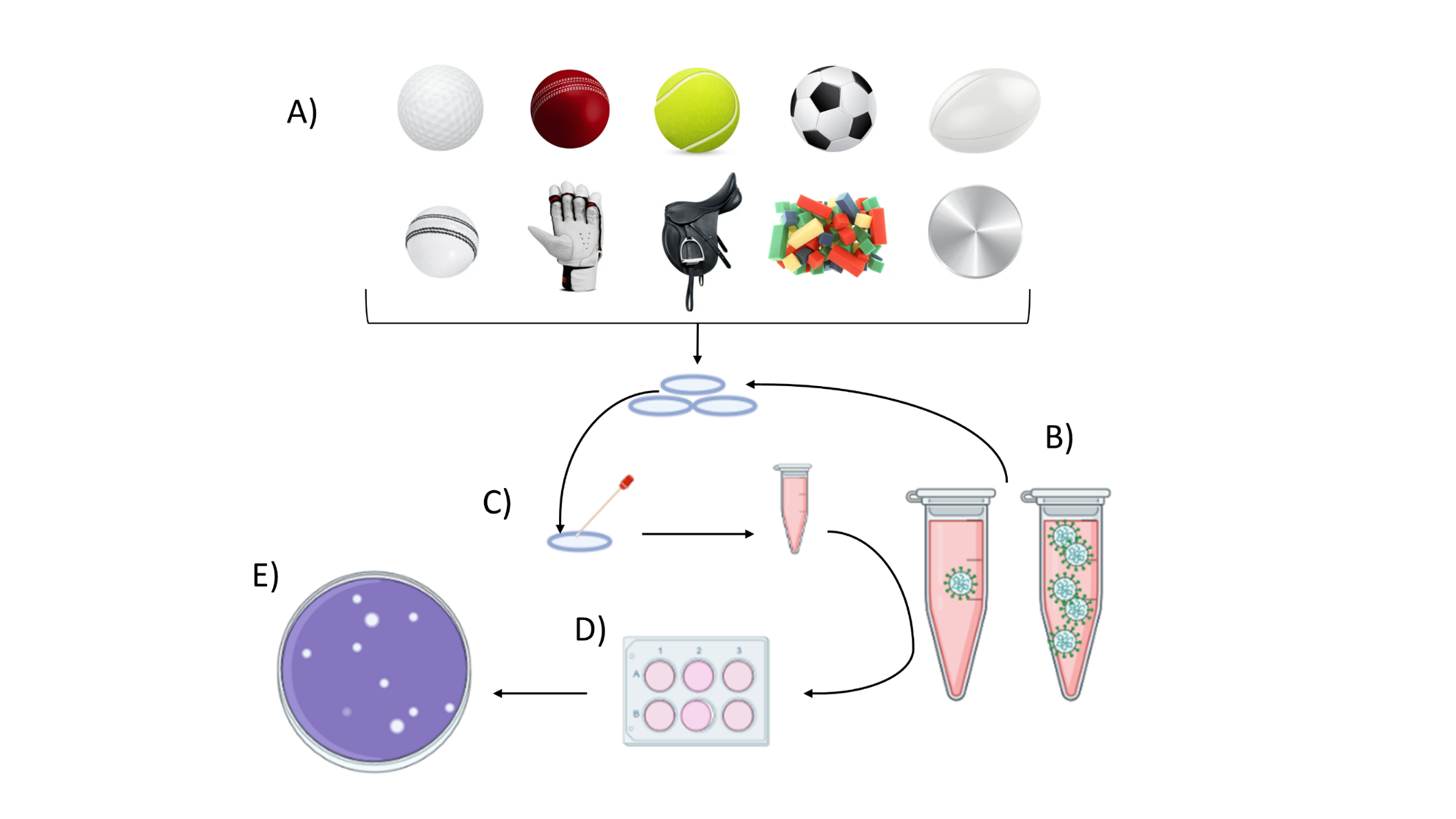


**Fig.S1.** Schematic of the experimental methods used in the study. Various sports equipment was collected, and triplicate 2cm disks were removed, per time point tested (A). A high (5.4x10^2^ PFU/ml) and low inoculum (5.4x10^4^ PFU/ml) of SARS-CoV-2 was added to the disks, which were dried at room temperature over the following time points; 1, 5, 15, 30, 90 minutes (B). The materials were then swabbed to retrieve live virus particles, which were then incubated onto Vero E6 cells (D), prior to staining for plaque enumeration (E)


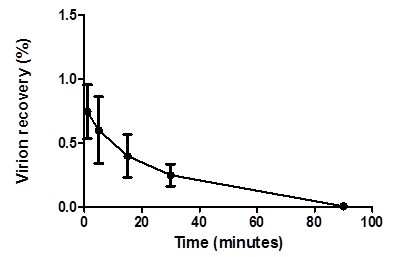


**Figure S2.** Proportion of virions recovered from high inoculum of 5.4x10^4^ from all materials at each time point. Error bars represent the standard error of the mean.
